# The inhibitory effect of word neighborhood size when reading with central field loss is modulated by word predictability and reading proficiency

**DOI:** 10.1038/s41598-020-78420-0

**Published:** 2020-12-11

**Authors:** Lauren Sauvan, Natacha Stolowy, Carlos Aguilar, Thomas François, Núria Gala, Frédéric Matonti, Eric Castet, Aurélie Calabrèse

**Affiliations:** 1 North Hospital, Marseille, France; 2 Mantu Lab, Amaris Research Unit, Sophia Antipolis, France; 3 UCLouvain, CENTAL (IL&C), Louvain-la-Neuve, Belgium; 4 CNRS UMR 7309, Aix-Marseille Univ., Aix-en-Provence, France; 5 Centre Monticelli Paradis d’Ophtalmologie, Marseille, France; 6 CNRS UMR 7290, Aix-Marseille Univ., Marseille, France; 7 Inria, Université Côte d’Azur, Sophia Antipolis, France

**Keywords:** Macular degeneration, Quality of life, Rehabilitation, Translational research, Human behaviour

## Abstract

For normally sighted readers, word neighborhood size (i.e., the total number of words that can be formed from a single word by changing only one letter) has a facilitator effect on word recognition. When reading with central field loss (CFL) however, individual letters may not be correctly identified, leading to possible misidentifications and a reverse neighborhood size effect. Here we investigate this inhibitory effect of word neighborhood size on reading performance and whether it is modulated by word predictability and reading proficiency. Nineteen patients with binocular CFL from 32 to 89 years old (mean ± SD = 75 ± 15) read short sentences presented with the self-paced reading paradigm. Accuracy and reading time were measured for each target word read, along with its predictability, i.e., its probability of occurrence following the two preceding words in the sentence using a trigram analysis. Linear mixed effects models were then fit to estimate the individual contributions of word neighborhood size, predictability, frequency and length on accuracy and reading time, while taking patients’ reading proficiency into account. For the less proficient readers, who have given up daily reading as a consequence of their visual impairment, we found that the effect of neighborhood size was reversed compared to normally sighted readers and of higher amplitude than the effect of frequency. Furthermore, this inhibitory effect is of greater amplitude (up to 50% decrease in reading speed) when a word is not easily predictable because its chances to occur after the two preceding words in a specific sentence are rather low. Severely impaired patients with CFL often quit reading on a daily basis because this task becomes simply too exhausting. Based on our results, we envision lexical text simplification as a new alternative to promote effective rehabilitation in these patients. By increasing reading accessibility for those who struggle the most, text simplification might be used as an efficient rehabilitation tool and daily reading assistive technology, fostering overall reading ability and fluency through increased practice.

## Introduction

Individuals with Central Field Loss (CFL) induced by maculopathy experience severely impaired functional vision, leading to major incapacitating reading deficits^1–3^. Reading speed being a strong determinant of overall quality of life in low-vision patients of all ages, there is a real societal need to help promote their reading performance through rehabilitation and assistive reading technology^4,5^. Such initiative requires a better characterization of the underlying factors involved in their overall reading deficit, which remain poorly understood. The influence of psycholinguistic factors, for instance, have been generally overlooked in the context of low vision. In 2016, Calabrèse et al. investigated eye movement characteristics when low-vision patients with CFL read short sentences and showed that specific disruptions in fixation patterns occurred in the presence of low-frequency words^6^. This result alone suggests that psycholinguistic factors may be an important determinant of reading performance for low vision, opening the way for further investigations and potential use of text simplification for the visually impaired.

**Figure 1:**
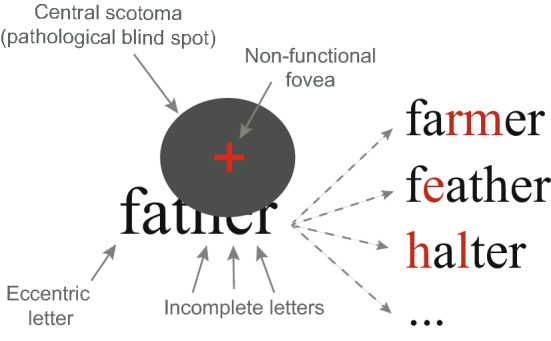
The presence of a central scotoma occludes part of the word “father”, leading to possible misidentifications, potentially due to: (1) incomplete letters (e.g., farmer); (2) eccentric letters, for which low acuity and increased crowding decrease identification performance (e.g., feather) or (3) both (e.g., halter). The overall lack of identification accuracy yields a greater need for linguistic inference.

Word frequency and word neighborhood size are some of the most important lexical factors known to affect reading in normal vision^7,8^. However, whether findings from normally sighted readers can be applied to low-vision individuals is not obvious. In the case of CFL, visual input is deteriorated because of blur, distortion and/or occluded letters, and access to text is only partial (Fig. 1)^9^. For instance, when reading the word “father” with a central blind spot, some letters are totally or partially occluded by the scotoma. In addition, complete letters that fall out of the scotoma must be identified with eccentric portions of the retina, yielding degraded letter identification due to low acuity and strong crowding^10^. Therefore, incomplete and/or eccentric letters may not be correctly identified, leading to many possible misidentifications (“farmer”, “feather”, “halter”, etc.). Since bottom-up visual input is not reliable, patients must rely much more on top-down linguistic inference than normally sighted readers^11–13^. For this reason, the effect of lexical factors on reading performance should be rather different in visually impaired readers than reported before with normally sighted individuals.

A first attempt to characterize this effect has been made recently by Stolowy et al. who inspected the effect of word frequency on the reading performance of CFL individuals reading sentences in French^14^. The clear-cut frequency effects they reported on word reading time validated the hypothesis that, as for normally sighted readers, low-frequency words tend to decrease reading speed with CFL patients. However, the amplitude of this effect was much larger (differences in the range of seconds) than it had been reported before with normal vision (range of milliseconds). More interestingly, the same effect of frequency was not observed for all pairs of synonyms. For instance, the high-frequency word “utiliser” [*use* in English] was read on average more slowly than its lower frequency synonym *“employer”* [*employ*]. Such observation suggests that frequency cannot be the only predictive linguistic factor of reading speed with CFL. This reasoning was a cornerstone for the present work that aims at investigating two other psycholinguistic factors extensively studied in normal reading: orthographic similarity and word predictability.

The most common measure of orthographic similarity in the psychological literature is Coltheart’s orthographic neighborhood size metric ‘N’^15^. N measures the number of close orthographical neighbors of a stimulus word and is defined as the total number of words that can be formed by changing one letter, while preserving letter positions. For example, “shore” has many neighbors, including chore, score and share*,* while “neighbor” has 0 neighbors. For skilled readers with normal vision, N (i.e., word neighborhood size) seems to have a facilitator effect on word recognition: the more neighbors, the faster a word is identified—although this effect is now often assumed to be task dependent and language dependent (see^16,17^ for reviews). It is possible that the visual constraint imposed by the presence of a central scotoma, hiding portions of the text (i.e., letters) and forcing to use eccentric vision (low resolution), may lead to a reverse effect of neighborhood size. The lack of high resolution coupled with missing visual information, would lead CFL readers to confuse one word with its orthographic neighbors, creating even more uncertainty for words with large neighborhood size. Therefore, we hypothesize that, unlike normal vision, word neighborhood size has a negative effect on reading speed with CFL (Hypothesis 1). This first hypothesis has recently received support through some preliminary work of ours^18^.

In addition to frequency and orthographic similarity, word predictability counts as one of the most influential processing factors during word recognition with normal vision^19^. The predictability of a given word in a sentence (based on the context induced by the preceding words) has been shown to influence processing speed by enabling readers to make forward inferences (i.e. predictions about the probable upcoming word). Thus, as shown by eye movement studies, highly predictable words: (1) are skipped more often, (2) are more likely to be identified within a single fixation and (3) yield overall shorter fixations during sentence reading^20,21^. In the context of CFL, word predictability should also play an additional role by reducing uncertainty when identifying a word with many confusable neighbors. In the sentence “You should go for a walk along the shore” for instance, even if the word “shore” has many potentially confusable neighbors, most of them (such as chore, score and share) may be easily ruled out based on semantic context and forward inferences. Therefore, we hypothesize that the amplitude of the neighborhood size effect is influenced by word predictability: the more predictable a word is (thanks to sentence context), the smaller the effect of neighborhood size should be (Hypothesis 2).

Finally, for normally sighted adult readers, word predictability does influence reading processing differently depending on individual reading skills^21,22^. Simply put, less skilled readers rely more on context for word identification than highly skilled readers do. Based on this result, we assume that results of CFL individuals should also be influenced by their reading proficiency. Therefore, we hypothesize that the interaction between neighborhood size and word predictability (see Hypothesis 2) depends on patients’ reading proficiency. Our prediction is that this interaction will be more pronounced for less proficient readers than for proficient ones (Hypothesis 3).

In short, the purpose of this work is twofold: (1) to test the hypothesis that word neighborhood size exerts an inhibitory effect on reading performance with CFL (Hypothesis 1; Analyses 1 and 2) and (2) to test whether this effect is modulated by word predictability and reading proficiency (Hypothesis 2 and 3; Analysis 3).

## Methods

### Participants

19 patients (13 females) were recruited from the Low-Vision Clinic of the La Timone Hospital (Marseille, France) between March and June 2017. All presented a bilateral central scotoma with a monocular acuity of 0.4 logMAR (i.e., 20/50 or 4/10) or worse in their better eye. Patients with ophthalmologic disorders other than maculopathy (e.g. glaucoma), cognitive disorders or reading disorders present before their visual impairment were not included. The following information was collected for each participant: age, etiology, lens status, disease onset (duration of maculopathy in years), field loss (central only vs. central + peripheral), low-vision rehabilitation history. Additional information was collected regarding their reading habits, including: current daily reading time (in minutes), current/former profession, whether they considered themselves heavy readers before their impairment. Recruited participants ranged in age from 32 to 89 years (mean ± SD = 75 ± 15). Mean best-corrected visual acuity was 0.81 ± 0.28 logMAR. Details of the participants’ demographic, visual and reading characteristics are given in Table 1. The research was approved by the Ethics Committee of the French Society of Ophthalmology (IRB 00008855 Société Française d’Ophtalmologie IRB#1) and carried out in accordance with the Code of Ethics of the World Medical Association (Declaration of Helsinki). Informed consent was obtained from all participants after complete explanation of the nature and possible consequences of the study.

**Table 1:**
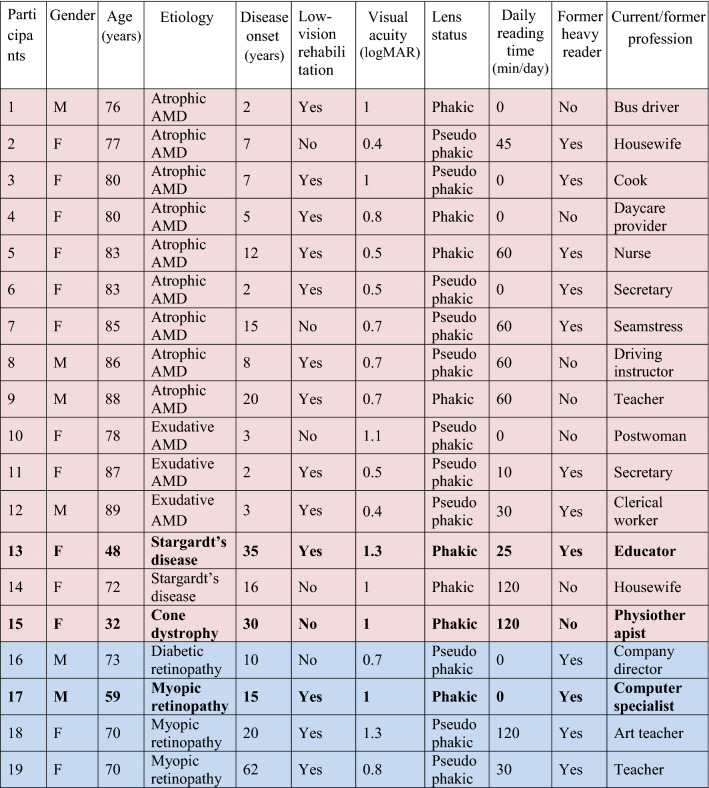
Participants’ demographic, visual and reading characteristics.

Visual acuity and lens status are given for the tested eye only. Participants with central scotoma only are highlighted in red. Participants with central scotoma and peripheral lesions are highlighted in blue. Participants who are still active workers are highlighted in bold font.

### Apparatus and stimuli

Experiments were run at 60 Hz with an LCD HP LE2201W monitor (full display area: 1680 × 1050 pixels; 47.4 × 29.6 cm). Stimuli (i.e. sentences) were generated with the PsychoPy library^23,24^ and presented on a 1400 × 1050 pixel window that subtended 56° × 42° at 40 cm. Sentences were aligned to the left and displayed in Courier (non-proportional font) in black on a white background. Print size was chosen optimally for each participant as the value of his/her critical print size, measured before testing with a French computerized version of MNREAD^25,26^. Reading was monocular (eye with better visual acuity) with an appropriate correction for near vision (wide-field Metrovision lenses). Monocular vision allows to better control for individual eye characteristics and was shown to yield similar reading performance compared to binocular vision in patients with binocular AMD^27^.

### Reading material

**Figure 2:**
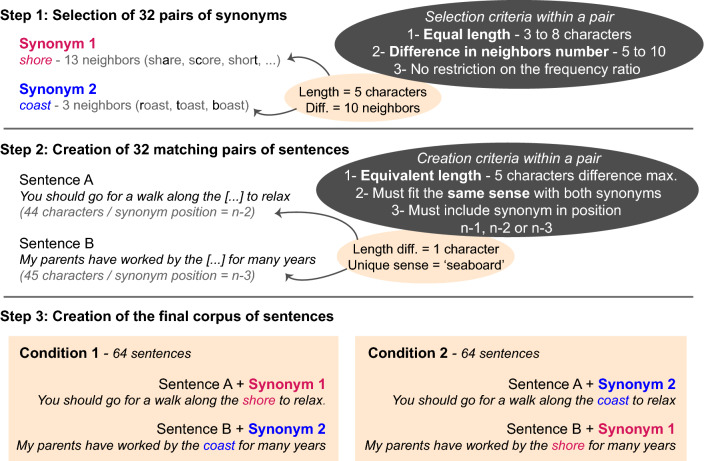
Creation of the reading material. Illustration of the reading material creation, using the synonym pair “*shore/coast*”. Both words in this example contain 5 characters. Their difference in number of neighbors is 10. Sentences A and B were created so either word of this synonym pair can fit in both sentences. In Condition 1, sentence A contains the target word 1 (‘shore’) and sentence B contains the target word 2 (‘coast’). In Condition 2, the pairing is reversed. Note that this example is given in English to ease understanding. See Supplementary Material for the complete set of French sentences created.

Reading material was created in French using ReSyf, a French lexicon with disambiguated and graded synonyms^28^ and Lexique3, a lexical database with word frequencies of standard written and oral French^29^. First, we created a pool of target words (Fig. 2—Step 1), by selecting 32 pairs of synonyms matching the following criteria: (1) equal length within a pair (from 3 to 8 characters); (2) difference in number of orthographic neighbors (N) comprised between 5 and 10 within a pair; (3) frequency ratio between a large neighborhood word and a its small neighborhood synonym could be either < 1 or > 1. Second, 32 pairs of matching sentences were created so that each word from a pair could fit within either sentence of the corresponding sentence pair (Fig. 2—Step 2). Three criteria were used: (1) all sentences had similar length (42 to 65 characters; mean ± SD = 54 ± 6), with a maximum difference of 5 characters within a pair; (2) pairs of sentences were specifically designed to fit the single and most frequent common sense of a synonym pair; (3) within each sentence, comprised of ‘n’ words, the target word could be located in any of these three locations: ‘n − 1’, ‘n − 2’, or ‘n − 3’. Target words were never presented in position ‘n’ to avoid any interference from a possible wrap-up effect^14,30^. At last, we created our final reading material by combining sentence pairs with their matching synonym pairs (Fig. 2—Step 3). In Condition 1, the first word of a pair was assigned to the first sentence of the corresponding pair, while the second word was assigned to the second sentence, thus creating 64 full sentences. In Condition 2, the “sentence—word” pairing was reversed to create a different set of 64 full sentences. These two experimental conditions allowed us to counterbalance any potential effect of the sentence structure and complexity (by randomly assigning participants to Condition 1 or 2), while providing two different measures of predictability for a single target word.

### Reading procedure and experimental design

**Figure 3:**
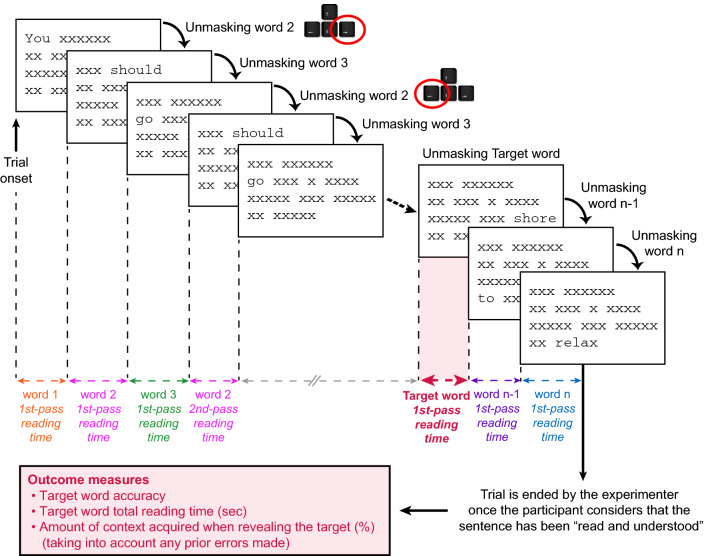
Self-paced Reading paradigm. Words are revealed one at a time by the participant, using keyboard presses. A press on the right arrow key will unmask the upcoming word (while masking the currently visible word) and a press on the left arrow key will unmask the previous word (while masking the currently visible word). For words unmasked only once (e.g. word 1), the total reading time equals the 1st-pass reading time. For words unmasked more than once (e.g. word 2), the total reading time is the sum of each individual reading time pass.

Sentences were presented within 4 blocks of 16 trials (8 pairs of sentences) each. Participants were randomly assigned to Condition 1 or 2 and read between two to four blocks, depending on their reading speed and level of fatigue. Sentences were displayed randomly within each block with non-cumulative self-paced reading, where sentences appear as a whole but with all words masked by strings of “ × ”^31–33^ (Fig. 3). As opposed to other reading paradigms specifically designed for low vision, such as “word mode”^34^, self-paced reading allows to present words individually while still maintaining a whole sentence view and therefore to remain closer to the visual constraints of natural reading (e.g., crowding). Participants were instructed to read each sentence aloud as quickly and accurately as possible while revealing each word one at a time using keyboard presses, with the possibility to unmask backward as many times as they wanted. When participants considered they had finished reading the sentence accurately (no matter which word was unmasked at that moment), they said the word “stop” and the experimenter stopped the trial. Prior testing, a training phase with short French proverbs was performed to familiarize the participant with the task and protocol. Reading accuracy (correct vs. incorrect) and total reading time (in seconds) were recorded for each target word. For words unmasked several times, reading time was obtained by summing the duration of all the unmasked periods.

### Measure of word predictability

For each target word, we estimated its percentage of occurrence following the two preceding words in the sentence based on a large corpus of French texts. To do so, we selected all series of three consecutive words (i.e., 3-grams or trigrams) in our reading material that ended with a target word. Using the Google Books Ngram Viewer resource^35^, we extracted for each of these 128 trigrams (64 sentences × 2 conditions) its percentage of occurrence in the ‘French 2012’ corpus, a corpus of 792 118 digitized books published in French between 1800 and 2008. This measure will be referred to as the ‘trigram occurrence’.

### Measures of reading proficiency

Prior to testing, each patient was asked to report the total duration of reading they performed on a typical day (in minutes per day; cf. Table 1). This value would include reading for both work and leisure, with or without visual aid systems, on all types of display (i.e., print, screen) and any kind of reading material (e.g., book, magazine, tag label, mail, recipe). Because of the large proportion of patients who reported to read 0 min/day (37%), this variable’s distribution was highly skewed and not suited to be used as a continuous variable. Therefore, we transformed it into the binary variable “Daily reading” (*yes or no*)*.* Patients who reported to read at least a few minutes each day were categorized as Daily reading—*yes;* The other patients who reported to read 0 min daily were categorized as Daily reading—*no*. In addition to this measure of current reading proficiency, patients were also grouped based on their self-reported literacy level prior impairment through the variable “Former heavy reader” (*yes or no*)*.*

### Statistical analysis

Statistical analyses were carried out using R, a free software environment for statistical computing and graphics^36^. Reading accuracy (i.e. binary variable) was analyzed by fitting a generalized linear mixed-effects model (GLME; Analysis 1), while reading time (i.e. continuous variable) was analyzed with linear mixed-effects models (LME; Analyses 2 and 3), both allowing the modeling of experimental designs with unbalanced repeated measures^37,38^. Models were constructed with either target word accuracy or target word reading time as the dependent variable. Several factors of interest (Table 2) were included as independent variables to inspect their effect on accuracy and reading time, as well as their potential interaction with each other. The random structure of all models included a random intercept for participants, assuming a different “baseline” performance level for each individual, as well as random intercept for each target word. Before analysis, variables of interest were inspected and transformed as follow to satisfy the assumptions of parametric statistical tests^39,40^: reading time and word frequency were transformed in natural logarithm (ln) units, trigram occurrence was transformed with the ordered quantile normalization and context amount was square-root transformed. Word frequency and word length were centered around their mean. Optimal model structures were assessed using the Akaike Information Criterion (AIC) and likelihood-ratio tests^41^. Significance of the fixed effects was estimated using z-values for the GLME model and t-values for the LME models. Z- and t-values larger than 2 were considered significant, corresponding to a 5% significance level in a two-tailed test^42,43^. In the Results section fixed-effects estimates are reported along with their z- and t-values and 95% confidence intervals^44^.

**Table 2:** Description of the different factors included as independent variables in the linear mixed-effects models.

Variable name	Unit/transformation	Centered	Description
**Target word characteristics**			
Neighborhood size (N)	–	–	Number of neighbors
Frequency (ln)	Natural log	Yes	Occurrences/million
Length	–	Yes	Number of characters
**Sentence characteristics**			
Trigram occurrence	Ordered quantile normalization	–	Percentage of occurrence for each trigram ending with a target word
**Participant’s individual characteristics**			
Age	Years	–	–
Disease onset	Years	–	–
Visual Acuity	LogMAR	–	–
Type of field loss	Central only Central + peripheral	–	–
Still reading daily	Yes No	–	–
Former heavy reader	Yes No	–	–

## Results

### Analysis 1: Effect of neighborhood size on reading accuracy

**Figure 4:**
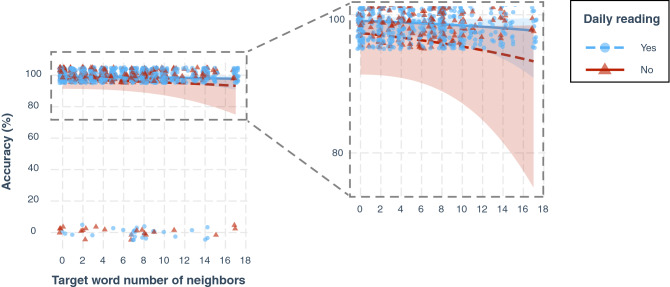
Effect of the neighborhood size (N) on word reading accuracy. Each data point represents a target word. Raw data points and fitted curves estimated by the GLME model are grouped according to reading proficiency: blue for participants reading daily, red for participants who quit daily reading activities. The right-side panel shows a zoomed view of the left plot between 70 and 100% accuracy.

On average, target words were read accurately 94% of the time, with individual variations ranging from 62 to 100% depending on patients. When all implemented in a GLME model, N, word frequency, word length and word predictability (expressed as trigram occurrence) showed no significant effect on accuracy (Table 3). As estimated by the model, percentage of accuracy for patients who continue reading on a daily basis was 99.1% (exp(4.716)/(1 + exp(4.716)) * 100) (z = 7.694; *p* < 0.001; 95%CI = [3.65; 6.17]). For patients who quit daily reading activities, percentage of accuracy was 97.3% (exp(4.716 − 1.126)/(1 + exp(4.716 − 1.126)) * 100). This 1.8% difference barely reached significance (z =  − 2.064; *p* = 0.039; 95%CI = [− 2.31; − 0.03]). Figure 4 shows the null effect of N on accuracy, for these two groups of participants.

**Table 3:** Fixed-effects estimates from the GLME model (analysis 1).

	Estimate	Standard Error	z-value	*p*-value	95% Confidence Interval
Intercept	4.716	0.613	7.694	< 0.001	[3.65; 6.17]
Word neighborhood size (N)	− 0.058	0.054	− 1.069	0.285	[− 0.17; 0.05]
Word frequency (ln)	0.147	0.147	1.003	0.316	[− 0.15; 0.46]
Word length	− 0.028	0.213	− 0.13	0.897	[− 0.47; 0.41]
Trigram occurrence	0.361	0.292	1.236	0.216	[− 0.21; 0.98]
***Daily reading*** ***No***	***− 1.126***	***0.545***	***− 2.064***	***0.039***	***[− 2.31; − 0.03]***

The dependent variable is ‘Target word reading accuracy’. The intercept estimate is the predicted value of the dependent variable when all independent variables are at 0 (continuous variables) or at their reference level (categorical variable). Reference level for the binary factor ”Daily reading” (representing reading proficiency) is ‘yes’. Factors showing a significant effect on reading accuracy are in bold font and highlighted in italic.

### Analysis 2: Effect of neighborhood size on reading time

**Figure 5:**
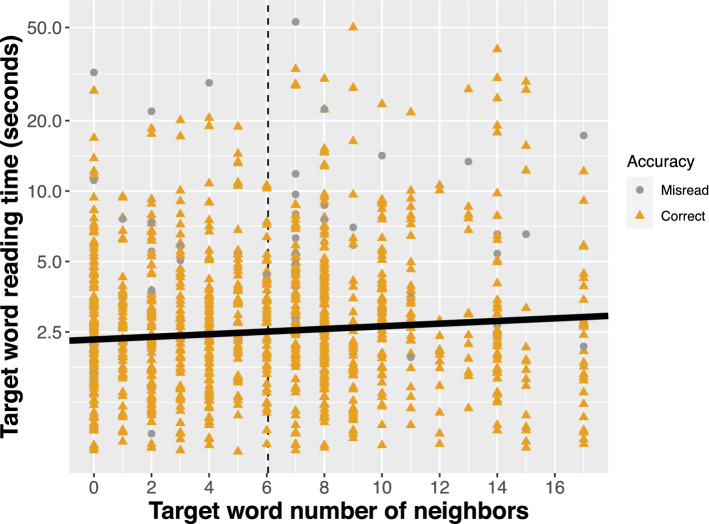
Effect of neighborhood size (N) on word reading time. Data for both well-read words (yellow triangles) and misread words (grey circles) are plotted but the fitted line extracted from the LME model (solid black) only considers the words correctly read. The black dotted line marks N mean value.

In this first LME model (Table 4), the respective effects of neighborhood size, word predictability and reading proficiency are estimated individually, without any interaction term, to test our Hypothesis 1. According to this simple model, words with zero neighbors were read on average in 2.3 s (exp(0.841)). Increasing the number of neighbors by 1 did increase reading time significantly, but moderately, by a factor of 1.01 (exp(0.013); t = 2.507, *p* = 0.013, 95%CI = [0.003; 0.023]; Fig. 5). In other words, increasing neighborhood size from 0 to 6 (the mean value in our pool of target words), increases reading time by a factor of 1.08 (exp(0.013)^6^), i.e. an 8% increase. Similarly, increasing neighborhood size from 0 to 10 (where most of our values lie), increases reading time by a factor of 1.14 (exp(0.013)^10^), i.e. a 14% increase. Word predictability (expressed as trigram occurrence) also showed a significant effect on reading time (t =  − 4.129, *p* < 0.001, [− 0.17; − 0.06]). Age, acuity, disease onset, type of field loss and former heavy reader (which were dropped from the final model) showed no significant effect on reading time and no significant interaction with word neighborhood size.

**Table 4:** Fixed-effects estimates from the simple LME model (analysis 2).

	Estimate	Standard Error	t-value	*p*-value	95% Confidence interval
Intercept	0.841	0.143	5.855	< 0.001	[0.55; 1.13]
***Word neighborhood size (N)***	***0.013***	***0.005***	***2.507***	***0.013***	**[0.003; 0.023]**
***Word frequency (ln)***	***− 0.047***	***0.013***	***− 3.419***	***< 0.001***	***[− 0.07; − 0.021]***
Word length	0.016	0.030	0.546	0.589	[**− **0.04; 0.08]
***Trigram occurrence***	***− 0.115***	***0.027***	***− 4.129***	***< 0.001***	***[− 0.17; − 0.06]***
Daily reading No	0.392	0.217	1.809	0.087	[**− **0.09; 0.87]

The dependent variable is log-transformed target word total reading time. The intercept estimate represents the log-transformed reading time when all factors included in the model are at their reference level (categorical variable) or at 0 (continuous variables). Reference level for the binary factor “Daily reading” is ‘yes’. Factors showing a significant effect on reading time are in bold font and highlighted in italic.

### Analysis 3: Effect of the interaction between neighborhood size, word predictability and reading proficiency on reading time

**Figure 6:**
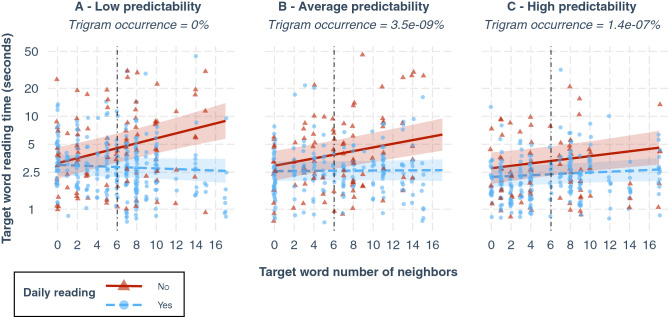
Effect of neighborhood size (N) on reading time, depending on word predictability and reading proficiency. Raw data points and fitted lines estimated by the LME model are grouped by reading proficiency: red for participants who quit reading on a daily basis, blue for participants still reading daily. Each subplot represents the effect of N for a different value of the trigram occurrence distribution, split into three equal-sized groups (i.e., terciles): (**A**) median of the lower tercile, (**B**) median of the middle tercile, (**C**) median of the upper tercile (**C**).

In this second LME model (Table 5), a 3-way interaction between neighborhood size, word predictability and reading proficiency was added to test our Hypotheses 2 and 3. According to this complex model, when trigram occurrence is at 0 (implying a highly infrequent trigram and low predictability; Fig. 6A), words with zero neighbors were read on average in 3.0 s (exp(1.099)) by patients who practice reading daily (Fig. 6A—blue dashed line). For this same group of readers, increasing the number of neighbors did not have a significant effect on reading time (t =  − 1.043, *p* = 0.298; 95%CI = [− 0.02; 0.01]; Fig. 6A—blue dashed line). For patients who quit daily reading activities, average reading time of words with zero neighbors was 3.14 s (exp(1.099 + 0.044)) and was not significantly different from the ‘daily reading’ group estimate (t = 0.188, *p* = 0.853, 95%CI = [− 0.42; 0.52]; Fig. 6A—red solid line). However, for these participants who stopped reading on a daily basis, increasing the number of neighbors by 1 did increase reading time significantly by a factor of 1.07 (exp(0.07); t = 5.22, *p* < 0.001, 95%CI = [0.04; 0.10]; Fig. 6A—red solid line). In other words, for low predictability, increasing neighborhood size from 0 to 6 (the mean value in our pool of target words), increases reading time by a factor of 1.52 (exp(0.07)^6^), i.e. a 52% increase. Similarly, increasing neighborhood size from 0 to 10 (where most of our values lie), increases reading time by a factor of 2.01 (exp(0.07)^10^), i.e. a 101% increase.

**Table 5:** Fixed-effects estimates from the complex LME model (analysis 3).

	Estimate	Standard error	t-value	*p*-value	95% Confidence interval
Intercept	1.099	0.136	8.09	< 0.001	[0.84; 1.37]
Word neighborhood size (N)	− 0.009	0.008	− 1.043	0.298	[− 0.02; 0.01]
***Word frequency (ln)***	***− 0.046***	***0.014***	***− 3.39***	***< 0.001***	***[− 0.07; − 0.02]***
Word length	0.013	0.028	0.48	0.633	[**− **0.04; 0.07]
***Trigram occurrence***	***− 0.162***	***0.049***	***− 3.24***	***0.002***	***[− 0.26; − 0.07]***
Daily reading No	0.044	0.234	0.188	0.853	[**− **0.42; 0.52]
***N:Daily reading*** ***No***	***0.07***	***0.013***	***5.22***	***< 0.001***	***[0.04; 0.10]***
Trigram occurrence: daily reading No	0.093	0.081	1.15	0.248	[− 0.06; 0.25]
N:Trigram occurrence: daily reading Yes	0.011	0.006	1.85	0.066	[− 4e−04; 0.02]
***N:Trigram occurrence: daily reading*** ***No***	***− 0.017***	***0.008***	***− 2.03***	***0.043***	***[− 0.03; − 8e−04]***

The dependent variable is log-transformed target word total reading time. The intercept estimate represents the log-transformed reading time when all factors included in the model are at their reference level (categorical variable) or at 0 (continuous variable). Reference level for the binary factor “Daily reading” is ‘yes’. Interactions are represented by the symbol “:”. Factors showing a significant effect on reading time are in bold font and highlighted in italic.

As trigram occurrence increases to an average value (Fig. 6B) and a high value (Fig. 6C), the effect of neighborhood size remains null for the ‘daily reading’ group (t = 1.85; *p* = 0.066; 95%CI = [− 4e−04; 0.02]; blue dashed lines). However, for the other group of patients who stopped practicing reading daily (red solid lines), the amplitude of the neighborhood size effect is significantly reduced by a factor of 1.02 (exp(0.017); t =  − 2.03; *p* = 0.043; 95%CI = [− 0.03; − 8e−04]) every time trigram occurrence increases by one unit. As given by a slopes post-hoc analysis, the amplitude of the effect for the group who quit daily reading (red solid line) was of 1.05 (exp(0.05); t = 5.39, *p* < 0.001) for average trigram occurrence values (Fig. 6B) and of 1.03 (exp(0.03); t = 2.77, *p* = 0.01) for highly frequent trigrams (Fig. 6C). In other words, for fairly frequent trigrams yielding average predictability, increasing neighborhood size from 0 to 10, increases reading time significantly by a factor of 1.65 (exp(0.05)^10^), i.e. a 65% increase, while the same increase in neighborhood size for highly frequent trigrams yielding high predictability, increases reading time significantly by a factor of only 1.35 (exp(0.03)^10^), i.e. a 35% increase.

Besides this significant 3-way interaction, word frequency also had a significant effect on reading time with a regression coefficient estimate of − 0.046 (t =  − 3.39, *p* < 0.001, 95%CI = [− 0.07; − 0.02]). As both reading time and frequency are expressed in natural log units, multiplying frequency (in original units) by 10 multiplies reading time (in original units) by 0.90 (10^−0.046^), i.e. a 10% decrease. We found no significant interaction between frequency and the ‘daily reading’ factor, nor between frequency and the number of neighbors. Word length had no significant effect on reading time (t = 0.48, *p* = 0.633, 95%CI = [− 0.04; 0.07]). Similarly, age, acuity, disease onset, type of field loss and former heavy reader (all dropped from the final model) showed no significant effect on reading time and no significant interaction with word neighborhood size.

## Discussion

The first goal of the present work was to test the hypothesis that the visual constraint imposed by the presence of a central scotoma leads to an inhibitory effect of neighborhood size during sentence reading. Therefore, we assessed the effect of word neighborhood size (N) on the reading performance of 19 patients with CFL, namely reading accuracy (Analysis 1) and reading time (Analysis 2). Our results show that N has no significant effect on accuracy, which ceils around 94% overall. On the other hand, we found a moderate inhibitory effect of N on reading time, with a 14% increase in word reading time (i.e., a 12% decrease in reading speed) when N goes from 0 to 10 neighbors (i.e., the range where most of our values lie). This result confirms our first hypothesis and builds up the recent report of a reversed neighborhood size effect for visually impaired individuals compared to normally sighted readers^18^.

Effects of orthographic neighbors on word identification have been extensively explored in readers with normal vision. Despite the many contradictory findings accumulated over the years, it is now accepted that the neighborhood size effect depends on the task and is modulated by the frequency of the neighbors themselves (i.e. the neighborhood frequency effect). Indeed, large neighborhood has consistently been reported to facilitate responses in a variety of tasks such as word naming^45^ and lexical decision^46,47^, but this facilitative effect seems to be restricted to low-frequency neighbors^48^. Despite their great interest to help understand the underlying mechanisms of lexical processing, these results are difficult to interpret in the context of our work with natural reading since they are restrained to isolated word identification.

Because semantic context may help decide between visually similar words, it is necessary to examine the effects of neighborhood (i.e., size and frequency) during natural reading in context to assess their influence on reading performance. To this end, Pollatsek et al. examined eye movement patterns of normally sighted readers during both a lexical decision task and silent reading, when target words varying in neighborhood size were embedded in neutral sentences^49^. Their overall conclusion was that, for silent reading, increasing the number of higher frequency neighbors had a clear inhibitory effect on word identification, whereas increasing the number of lower frequency neighbors may have a weak facilitative effect. In the present work, reading material was created without controlling for neighborhood frequency, but a post-hoc analysis revealed that most of our target words (82%) had a majority of low-frequency neighbors (from 60 to 100%; mean = 83 ± 14%). Based on this distribution, results from normal vision would predict a weak facilitative effect of neighborhood size on reading performance^49^. On the contrary, we found a reverse effect of neighborhood size, with a weak inhibitory effect. This result confirms our assumption that under degraded visual conditions, the lack of complete stimulus information will have more of an effect on words that are visually similar to many others than on words with few neighbors. It is likely that in the interest of time performance, readers may infer a word by guessing one of its high-frequency neighbors. However, because of the incongruousness between this guess and the overall sentence meaning, processing would persist until a better match, that fits both the visually identified letters and the meaning, is found. This would explain why reading accuracy remained very high among our participants, at the expense of reading time.

The second goal of the present work was to test the hypotheses that, with CFL, the effect of neighborhood size is modulated by both word predictability and reading proficiency. Therefore, we assessed the word neighborhood size effect through a 3-way interaction, including a measure of predictability (trigram occurrence) and proficiency (daily reading—*yes or no*) (Analysis 3). First, we found that the inhibitory effect of N is modulated by word predictability: the more familiar a sequence of words is, the weaker the effect of neighborhood size on the last word identification time. This result confirms our second hypothesis that the amplitude of the neighborhood size effect is influenced by word predictability.

However, we found that this is only true for the less proficient readers, who have stopped reading on a daily basis, confirming our third hypothesis that the interaction between neighborhood size and word predictability depends on patients’ reading proficiency. In short, we found that for the less proficient readers reaching a low-predictable word in a sentence, reading speed decreases by up to 50% (101% increase in reading time) when the number of neighbors goes from 0 to 10. As predictability increases, the amplitude of this effect lessens gradually, with 39% decrease in reading speed for average predictability (65% increase in reading time) and 26% decrease in reading speed for high predictability (35% increase in reading time). For proficient readers who reported to retain a daily leisure activity of reading however, even for a few minutes each day, the effect of N remains null, regardless of the word predictability. This result, close to what has been reported with normally sighted readers^49^, leads us to the conclusion that practice can help minimize the adverse effect of ambiguity induced by orthographic similarity when visual input is degraded and access to text is only partial because of maculopathy.

These results are particularly relevant in the context of low-vision rehabilitation, as they reinforce the need to provide patients with individualized readaptive care of functional vision, in order to help maintain daily reading practice. More importantly, our results suggest that text simplification might be a powerful way to leverage text accessibility for low-vision patients. Text simplification is the process of reducing the linguistic complexity of a text, while still retaining the original information and meaning^50,51^. Its main goal is to make a text more accessible to people with low literacy^52^ or individuals with reading disorders (e.g., aphasia^53^, dyslexia^54^). For the first time, our results show that it could also be used efficiently to improve low-vision rehabilitation, especially for the most impaired patients, who have stopped reading on a daily basis (36.8% in our population sample). For these less proficient readers, substituting complex words (i.e., words with many orthographic neighbors) with synonyms that have less neighbors and equal or higher frequency should reduce reading difficulty. Therefore, using simplified texts with increased accessibility as rehabilitation training material might help (1) improve overall reading ability and fluency, while (2) fostering the long-term motivation necessary to resume daily reading practice. Text simplification could then be used daily as an efficient reading aid (made available through tablets, e-readers, or web plugins) to keep practicing reading at home and enhance everyday reading performance.

As a side note, we would like to point to the fact that our results were obtained using the occurrence percentage of trigrams (3-grams, i.e., sequence of three words) in the French literature. It is worth mentioning that we also ran the analysis with other n-gram values, namely 2- and 4-grams and that neither of them showed significant effect. Since most of our target words were common nouns (89%; against 9.4% of adjectives and 1.6% of verbs), they were most likely preceded by an article that did not convey meaningful information. Therefore, an analysis based on 2-gram prediction was not likely to be meaningful. On the other hand, we expected 4-gram analysis to be highly significant. However, because we created our reading material so that target words were not too predictable, 77% of our 4-gram were highly infrequent (less than 40 occurrences across the ‘French 2012’ corpus) and rated at 0 percent occurrence by Google Ngram. We suspect that the absence of significant effect with 4-grams is due to this highly skewed distribution towards 0.

Overall, the present work presents some limitations that should be considered in future investigations of neighborhood effects on low vision. First, the range of participants’ age and disease onset should be expanded to better represent the full spectrum of adaptation exhibited following early and late onset CFL. It is possible that the absence of effect reported here may be due to our highly skewed distribution, with 16 participants between 70 and 89, against only 3 young individuals (aged 32, 48 and 59). Second, future investigations should also include measures of microperimetry (size and shape of the scotoma, fixation eccentricity, etc.) to take into account individual vision loss characteristics. Third, neighborhood frequency should be gauged thoroughly when designing the reading material in order to better control for its effect when assessing the effect of neighborhood size. Second, the definition of orthographic neighbor used in this work is letter-position-specific and length-dependent^15^. Given that letter position uncertainty is a crucial factor limiting peripheral word recognition, and reading without central vision in general^55^, Coltheart’s definition should probably be extended to include letter transposition (e.g., trial and trail), addition (e.g. trial and tribal) and deletion (e.g. trial and rial) in order to encompass a larger number of highly similar words. Finally, the results presented here should be interpreted cautiously in the context of reading under natural conditions, since they were obtained with a paradigm that does not allow word skipping, forcing participants to read each single word of a sentence^56^. Additionally, reading performance was measured with monocular vision, allowing to control for specific eye characteristics (e.g., lens status). Such approach is critical in research settings, but may not always mimic actual clinical conditions, where patients would read with one or two eyes, based on their own preference.

## Supplementary information

Supplementary Information.

## References

[1] Brown JC, Goldstein JE, Chan TL, Massof R, Ramulu P, Low Vision Research Network Study Group (2014). Characterizing functional complaints in patients seeking outpatient low-vision services in the United States. Ophthalmology.

[2] Kanonidou E (2011). Reading performance and central field loss. Hippokratia.

[3] Chung STL (2020). Reading in the presence of macular disease: a mini-review. Ophthal. Physiol Opt..

[4] Murro V, Sodi A, Giacomelli G, Mucciolo DP, Pennino M, Virgili G (2017). Reading ability and quality of life in stargardt disease. Eur. J. Ophthalmol..

[5] Pondorfer SG, Terheyden JH, Heinemann M, Wintergerst MWM, Holz FG, Finger RP (2019). association of vision-related quality of life with visual function in age-related macular degeneration. Sci. Rep..

[6] Calabrèse A, Bernard J-B, Faure G, Hoffart L, Castet E (2016). Clustering of eye fixations: a new oculomotor determinant of reading speed in maculopathy. Invest. Ophthalmol. Vis. Sci..

[7] Leroy G, Kauchak D (2014). The effect of word familiarity on actual and perceived text difficulty. J. Am. Med. Inform. Assoc..

[8] Adelman JS, Brown GDA (2007). Phonographic neighbors, not orthographic neighbors, determine word naming latencies. Psychon. Bull. Rev..

[9] Taylor DJ, Edwards LA, Binns AM, Crabb DP (2018). Seeing it differently: self-reported description of vision loss in dry age-related macular degeneration. Ophthal. Physiol. Opt..

[10] Chung STL (2007). Learning to identify crowded letters: does it improve reading speed?. Vis. Res..

[11] Bullimore MA, Bailey IL (1995). Reading and eye movements in age-related maculopathy. Optom. Vis. Sci..

[12] Fine EM, Peli E (1996). The role of context in reading with central field loss. Optom. Vis Sci..

[13] Legge GE, Klitz TS, Tjan BS (1997). Mr. Chips: an ideal-observer model of reading. Psychol. Rev..

[14] Stolowy N, Calabrèse A, Sauvan L, Aguilar C, François T, Gala N (2019). The influence of word frequency on word reading speed when individuals with macular diseases read text. Vis. Res..

[15] Coltheart M, Davelaar E, Jonasson JE, Besner D, Dornio S (1977). Access to the internal lexicon. Attention and Performance VI.

[16] Andrews S (1997). The effect of orthographic similarity on lexical retrieval: resolving neighborhood conflicts. Psychon. Bull. Rev..

[17] Perea M, Martínez E (2000). The effects of orthographic neighborhood in reading and laboratory word identification tasks. Psicológica.

[18] Sauvan, L. *et al.* Text simplification to help individuals with low vision to read more fluently. In *Workshop Tools and Resources to Empower People with Reading Difficulties (READI) at International conference on Language Resources and Evaluation*. 27–32 (2020).

[19] Rayner K (1998). Eye movements in reading and information processing: 20 years of research. Psychol. Bull..

[20] Balota DA, Pollatsek A, Rayner K (1985). The interaction of contextual constraints and parafoveal visual information in reading. Cogn. Psychol..

[21] Hawelka S, Schuster S, Gagl B, Hutzler F (2015). On forward inferences of fast and slow readers. An eye movement study. Sci. Rep..

[22] Ashby J, Rayner K, Clifton C (2005). Eye movements of highly skilled and average readers: differential effects of frequency and predictability. Q. J. Exp. Psychol. A.

[23] Peirce JW (2007). PsychoPy–psychophysics software in Python. J. Neurosci. Methods.

[24] Peirce JW (2009). Generating stimuli for neuroscience using PsychoPy. Front. Neuroinform..

[25] Calabrèse A, Bernard J-B, Faure G, Hoffart L, Castet E (2014). Eye movements and reading speed in macular disease: the shrinking perceptual span hypothesis requires and is supported by a mediation analysis. Invest. Ophthalmol. Vis. Sci..

[26] Calabrèse, A., Mansfield, J. S., & Legge, G. E. *mnreadR, an R Package to Analyze MNREAD Data. version 2.1.3* (accessed December 2020). https://CRAN.R-project.org/package=mnreadR (2019).

[27] Kabanarou SA, Rubin GS (2006). Reading with central scotomas: is there a binocular gain?. Optom. Vis. Sci..

[28] Billami, M., François, T., & Gala, N. ReSyf: a French lexicon with ranked synonyms. In *Proceedings of the 27th Conference on Computational Linguistics (COLING 2018), Santa Fe, USA*, 2570–2581 (accessed December 2020). https://cental.uclouvain.be/resyf/ (2018).

[29] New B, Ferrand L, Pallier C, Brysbaert M (2006). Reexamining the word length effect in visual word recognition: new evidence from the English Lexicon Project. Psychon. Bull. Rev..

[30] Just MA, Carpenter PA (1980). A theory of reading: from eye fixations to comprehension. Psychol. Rev..

[31] Aaronson D, Scarborough HS (1976). Performance theories for sentence coding: some quantitative evidence. J. Exp. Psychol. Hum. Percept. Perform..

[32] Mitchell DC, Green DW (1978). The effects of context and content on immediate processing in reading. Q. J. Exp. Psychol..

[33] Just MA, Carpenter PA, Woolley JD (1982). Paradigms and processes in reading comprehension. J. Exp. Psychol. Gen..

[34] Wallis S, Yang Y, Anderson SJ (2018). Word Mode: a crowding-free reading protocol for individuals with macular disease. Sci. Rep..

[35] Michel J-B (2011). Quantitative analysis of culture using millions of digitized books. Science.

[36] R Core Team. *R: A Language and Environment for Statistical Computing*. Vienna, Austria: R Foundation for Statistical Computing (accessed December 2020). https://www.R-project.org/ (2018).

[37] Bolker BM, Brooks ME, Clark CJ, Geange SW, Poulsen JR, Stevens MH (2009). Generalized linear mixed models: a practical guide for ecology and evolution. Trends Ecol. Evol..

[38] Cheng J, Edwards LJ, Maldonado-Molina MM, Komro KA, Muller KE (2010). Real longitudinal data analysis for real people: building a good enough mixed model. Stat. Med..

[39] Tabachnick BG, Fidell LS, Ullman JB (2007). Using Multivariate Statistics.

[40] Howell DC (2009). Statistical Methods for Psychology.

[41] Zuur AF, Ieno EN, Elphick CS (2010). A protocol for data exploration to avoid common statistical problems. Methods Ecol. Evol.

[42] Baayen RH, Davidson DJ, Bates DM (2008). Mixed-effects modeling with crossed random effects for subjects and items. J. Mem. Lang..

[43] Gelman A, Hill J (2007). Data Analysis Using Regression and Multilevel/Hierarchical Models.

[44] Bates D, Mächler M, Bolker B, Walker S (2015). fitting linear mixed-effects models using lme4. J. Stat. Softw..

[45] Andrews S (1989). Frequency and neighborhood effects on lexical access: activation or search?. J. Exp. Psychol. Learn. Mem. Cognit..

[46] Sears CR, Hino Y, Lupker SJ (1995). Neighborhood size and neighborhood frequency effects in word recognition. J. Exp. Psychol. Hum. Percept. Perform..

[47] Forster KI, Shen D (1996). No enemies in the neighborhood: absence of inhibitory neighborhood effects in lexical decision and semantic categorization. J. Exp. Psychol. Learn. Mem. Cogn..

[48] Carreiras M, Perea M, Grainger J (1997). Effects of orthographic neighborhood in visual word recognition: cross-task comparisons. J. Exp. Psychol. Learn. Mem. Cogn..

[49] Pollatsek A, Perea M, Binder KS (1999). The effects of "neighborhood size" in reading and lexical decision. J. Exp. Psychol. Hum. Percept. Perform..

[50] Siddharthan A (2014). A survey of research on text simplification. Int. J. Appl. Linguist..

[51] Saggion H (2017). Automatic text simplification. Synth. Lect. Hum. Lang. Technol..

[52] Candido, Jr., A. *et al*. Supporting the adaptation of texts for poor literacy readers: a text simplification editor for Brazilian Portuguese. In *Proceedings of the Fourth Workshop on Innovative Use of NLP for Building Educational Applications* 34–42 (2009).

[53] Carroll, J. *et al*. Simplifying text for language-impaired readers. In *Proceedings of the 9th Conference of the European Chapter of the Association for Computational Linguistics (EACL)* 269–270 (1999).

[54] Rello, L., *et al*. DysWebxia 2.0!: more accessible text for people with Dyslexia. In *Proceedings of the 10th International Cross-Disciplinary Conference on Web Accessibility* **25**, 1–2 (2013).

[55] Bernard J-B, Castet E (2019). The optimal use of non-optimal letter information in foveal and parafoveal word recognition. Vis. Res..

[56] Albrengues C, Lavigne F, Aguilar C, Castet E, Vitu F (2019). Linguistic processes do not beat visuo-motor constraints, but they modulate where the eyes move regardless of word boundaries: evidence against top-down word-based eye-movement control during reading. PLoS ONE.

