# Supplementary material for: The inhibitory effect of word neighborhood size when reading with central field loss is modulated by word predictability and reading proficiency
Source: Sci Rep. 2020 Dec 11;10:21792. doi: 10.1038/s41598-020-78420-0 (PMC7733451; doi:10.1038/s41598-020-78420-0)
Supplement: Supplementary file 1 — Supplementary Information. [file 41598_2020_78420_MOESM1_ESM.docx]

**The inhibitory effect of word neighborhood size when reading with central field loss is modulated by word predictability and reading proficiency**

**Authors:** Lauren Sauvan^1^, Natacha Stolowy^1^, Carlos Aguilar^2^, Thomas François^3^, Núria Gala^4^, Frédéric Matonti^5^, Eric Castet^6^, Aurélie Calabrèse^7*^

**Affiliations:**

^1^ North Hospital, Marseille France

^2^ Mantu Lab, Amaris Research Unit, Sophia Antipolis, France

^3^ UCLouvain, CENTAL (IL&C), Louvain-la-Neuve, Belgique

^4^ Aix-Marseille Univ., CNRS UMR 7309, Aix-en-Provence, France

^5^ Centre Paradis Monticelli, Marseille, France

^6^ Aix-Marseille Univ., CNRS UMR 7290, Marseille, France

^7^ Université Côte d’Azur, Inria, France

**Corresponding author:**

Aurélie Calabrèse (AC)

aurelie.calabrese@inria.fr

2004 Route des Lucioles, 06902 Valbonne, France

SUPPLEMENTARY MATERIAL

Mon ami nous parlait de son **métier (boulot)** passionnant

Il ne s'ennuyait pas car c'était un **boulot (métier)** prenant

Il était aimé de tous, c'était le **chef (boss)** de l'usine

Antoine a toujours voulu être le **boss (chef)** du groupe

Je suis resté bloqué car le **loquet (verrou)** ne s'ouvrait plus

Si j'étais toi je ferais installer un **verrou (loquet)** sur la porte

Tu devrais aller faire une **marche (balade)** pour te détendre

Nous avons aimé cette belle **balade (marche)** dans les bois

La semaine prochaine il faudra faire un **arrêt (halte)** chez mamie

En rentrant nous avons fait une **halte (arrêt)** à la boulangerie

Il a finalement réussi à trouver une **faille (brèche)** dans le système

Ils sont entrés par une petite **brèche (faille)** à travers le mur

On marchait dans de la **boue (vase)** c'était désagréable

Il fallait traverser de la **vase (boue)** pour y accéder

Elle a longtemps travaillé avec un autre **type (gars)** du bureau

Chaque samedi il vendait des poulets avec un **gars (type)** du village

Avec sa voiture décapotable il aime **frimer (crâner)** en ville

Elle est toujours en train de **crâner (frimer)** avec sa robe

Ne me dis pas que le chien a encore mangé ma **godasse (soulier)** en cuir

Ce matin en rangeant j'ai trouvé mon **soulier (godasse)** sous le lit

Tu devrais aller te mettre au **repos (calme)** dans la chambre

Ça m'a fait du bien ce séjour au **calme (repos)** chez ma mère

Il a travaillé de longues années pour devenir un **expert (savant)** réputé

Je lui ai demandé son avis car c'est un **savant (expert)** dans ce domaine

Nous pensons qu'il s'agit d'un **germe (virus)** plutôt agressif

Elle n'est pas sortie car elle avait un **virus (germe)** dangereux

Faites attention, vous devez marcher sur le **bord (côté)** de la route

Les enfants se mettaient sur le **côté (bord)** pour laisser passer

J'étais la seule déguisée hier soir, c'était **marrant (amusant)** mais gênant

Ils se sont revus par hasard, c'est **amusant (marrant)** cette coïncidence

Ce serait gentil de vous mettre bien au **centre (milieu)** de l'écran

Il tira de toutes ses forces depuis le **milieu (centre)** du terrain

Tous les vendredis midi je vais au **bar (pub)** du village

La semaine dernière il m'a invitée au **pub (bar)** du coin

Si tu vas jusqu'à la falaise tu vois une **baie (anse)** sublime en bas

J'aimais marcher jusqu'à cette **anse (baie)** à l'abri des touristes

Au musée nous avons vu un ancien **char (tank)** très abîmé

On nous a laissé grimper dans un **tank (char)** de l'époque

Au milieu de la cour il y avait une **botte (gerbe)** de blé

Pour la décoration je mettrai une **gerbe (botte)** de fleurs

Elle portait toujours un **large (grand)** manteau en hiver

Devant la maison il y avait un **grand (large)** patio

Avec le brouillard ils n'ont pas vu la **jetée (digue)** devant eux

Chaque dimanche il marchait jusqu'à la **digue (jetée)** et revenait

Mon frère ne travaille pas, c'est un **feignant (flemmard)** depuis toujours

Il ne fallait pas se plaindre ni être un **flemmard (feignant)** pour réussir

Le patron n'a pas pu obtenir un **délai (durée)** acceptable

Ils peuvent me rembourser avec une **durée (delai)** convenable

Il a freiné d'un coup pour éviter le **bus (car)** qui passait

Je l'ai rencontré dans le **car (bus)** la semaine dernière

Pour valider ce certificat il faut le **cachet (tampon)** du médecin

Vous êtes sensé mettre un **tampon (cachet)** sur ces documents

Ça me brûlait depuis trois jours, j'avais une **saleté (crasse)** dans l'œil

Lorsque nous sommes entrés, il y avait de la **crasse (saleté)** partout

Je te préviens car cet objet est **cassant (fragile)** et j'y tiens

Je l'ai hérité de ma mère il est **fragile** **(cassant)** mais très utile

Je souhaite refaire ma cuisine avec cette **nuance (teinte)** de bleu

On distingue sur tous ses tableaux une **teinte (nuance)** particulière

Ils ont passé la journée dans la même **salle (pièce)** sans se parler

Pouvez-vous déposer ces affaires dans la **pièce (salle)** du fond

Pierre est énervant, il est **avare (radin)** comme son père

Il devenait de plus en plus **radin (avare)** avec le temps

Olivier a fait refaire sa cuisine et il est **content (heureux)** du résultat

Il l'a trouvé sur une brocante, il était **heureux (content)** de sa trouvaille
